# Sperm Morphology in Two House Mouse Subspecies: Do Wild-Derived Strains and Wild Mice Tell the Same Story?

**DOI:** 10.1371/journal.pone.0115669

**Published:** 2014-12-26

**Authors:** Jana Albrechtová, Tomáš Albrecht, Ludovít Ďureje, Vincent A. Pallazola, Jaroslav Piálek

**Affiliations:** 1 Research Facility Studenec, Institute of Vertebrate Biology, Academy of Sciences of the Czech Republic, Brno, Czech Republic; 2 Department of Zoology, Faculty of Science, Charles University in Prague, Prague, Czech Republic; 3 Department of Ecology and Evolutionary Biology and Museum of Zoology, University of Michigan, Ann Arbor, MI, United States of America; University of Massachusetts, United States of America

## Abstract

Being subject to intense post-copulatory selection, sperm size is a principal determining component of male fitness. Although previous studies have presented comparative sperm size data at higher taxonomic levels, information on the evolution of sperm size within species is generally lacking. Here, we studied two house mouse subspecies, *Mus musculus musculus* and *Mus musculus domesticus*, which undergo incipient speciation. We measured four sperm dimensions from cauda epididymis smears of 28 wild-caught mice of both subspecies. As inbred mouse strains are frequently used as proxies for exploring evolutionary processes, we further studied four wild-derived inbred strains from each subspecies. The subspecies differed significantly in terms of sperm head length and midpiece length, and these differences were consistent for wild mice and wild-derived strains pooled over genomes. When the inbred strains were analyzed individually, however, their strain-specific values were in some cases significantly shifted from subspecies-specific values derived from wild mice. We conclude that: (1) the size of sperm components differ in the two house mouse subspecies studied, and that (2) wild-derived strains reflect this natural polymorphism, serving as a potential tool to identify the genetic variation driving these evolutionary processes. Nevertheless, we suggest that more strains should be used in future experiments to account for natural variation and to avoid confounding results due to reduced variability and/or founder effect in the individual strains.

## Introduction

In promiscuous species, sperm size, as determined by male-male sperm competition and cryptic female choice [1]-[3], is a principal component of male fitness. Comparative data between more distantly related species (typically involving single or a few individuals per species) reveal variation in sperm size reaching up to three orders of magnitude among animal taxa and over 12-fold in mammals [4]-[10]. At the opposite end of the evolutionary spectrum, population-based studies focused on single species have found that higher levels of promiscuity are associated with longer sperm and decreased sperm size variation among males [11]-[13]. The house mouse represents a suitable model for studying sperm size evolution in a taxon at an intermediate level of divergence. Two house mouse subspecies, *Mus musculus musculus* (Mmm) and *Mus musculus domesticus* (Mmd), diverged about 350,000 years ago [14] and colonized Europe with the spread of agriculture [15]. In regions where their populations abut, these subspecies form a long, narrow secondary hybrid zone stretching from Norway to the Black Sea [16], [17]. For most of their divergence time, the two species' ranges were separated, allowing them to accumulate numerous genetic incompatibilities [18]. Recent studies have suggested that traits related to sperm quality and function, namely sperm number and sperm velocity, may be implicated in the maintenance of the house mouse hybrid zone (HMHZ) [19], [20]. Identification of spermatogenesis as a male fitness component with rapidly evolving incompatibilities led us to ask whether sperm size differentiation occurred simultaneous to mouse subspecific divergence.

Previous intrasubspecific studies relied on the presence of a variety of classical laboratory inbred strains (CLS) with a long-standing history of inbreeding, maintaining characteristic individual phenotypes [21]. Comparison of various classical strains revealed substantial strain-specific variation in sperm head dimension and in the incidence of head abnormalities; the inter-strain variation observed is explained by genetic differences among these traits - e.g. [22]-[24]. The observed sperm size differentiation is, however, difficult to extrapolate into an evolutionary pattern, as the classical strains are mixtures of three mouse genomes with limited, non-randomly distributed haplotype diversity [25].

To avoid the confounding factors mentioned above, wild-derived strains (WDS) can be a suitable substitution for CLS in understanding the evolutionary forces that shape sperm size. For example, Firman and Simmons (2010) [26] studied selection lines derived from WDS of Mmd (Mmd-WDS) and identified substantial, intrasubspecific variation in sperm midpiece length. This may suggest that variation also exists in natural populations. Although sperm size may be a key predictor for fertilization success in promiscuous species such as mice [26]-[29], only Mmd-WDS have been studied previously, and studies on wild mice in this context have not yet been published.

In this study, we measured four sperm components in wild mice and WDS from European allopatric populations that represent both house mouse subspecies. We focused on the (i) detection of intersubspecific differences in sperm size components and (ii) comparison of sperm phenotypic variability of wild-derived strains and wild individuals. Specifically, by decomposing phenotypic variation within and between compared groups, we aimed to determine whether WDS might be utilized to study the genetic basis of intra- and intersubspecific sperm differentiation, and furthermore to model evolutionary processes that affect sperm phenotypes.

## Materials and Methods

Wild mice from both Mmm (N = 28) and Mmd (N = 28) subspecies were live-trapped in several European allopatric populations from 2005 – 2011 (see Table 1 for their position). The subspecific origin of each male was based on geographic position of the locality relative to the position of the house mouse hybrid zone [16]. To confirm subspecific status, DNA was isolated from alcohol-preserved spleens and analyzed for the presence of diagnostic alleles at eight markers. The primers and PCR conditions for individual loci followed the protocol described earlier: *Bam*H I restriction site in the *Nd1* gene in mtDNA [30]; 18-bp deletion within the Y-linked Zink finger protein 2 gene (*Zfy2*) [31]; SINE B1 insertion in the Bruton agammaglobulinemia tyrosine kinase gene (*Btk*) and *Tsx* on the X chromosome [31], [32]; SINE B2 insertion 3′ downstream the *Syap1* gene [33]; LINE1 insertion *XL1_332L07* (*X332*), the SINE B2 insertion *XB2_347N11* (*X347*), and *X65* [34]. According to NCBI Build 38 (http://www.ncbi.nlm.nih.gov/), physical positions of the X-linked markers are: 17.13, 51.79, 73.77, 103.41, 135.54 and 161.57 Mb for *X332*, *X347, X65, Tsx, Btk* and *Syap1*, respectively.

**Table 1:** Position and genotypes of wild house mice and two Mmd-derived strains: SCHEST and SIT.

			Coordinates	mtDNA	Y-linked	X-linked markers					
Mouse	Locality	Country	Latitude/Longitude	*Bam*H I	*Zfy2*	*X332*	*X347*	*X65*	*Tsx*	*Btk*	*Syap1*
SK112	Feldkirch	Austria	50°15′/40°90′	d	d	m	d	d	d	d	d
SK1344	Sinj	Croatia	45°40′/15°40′	d	d	d	d	d	d	d	d
ST7601	Brouzet-lès-Quissac	France	45°50′/05°60′	d	d	d	d	d	d	d	d
SK1514	St-Jean-et-Royans	France	45°00′/05°15′	d	d	d	d	d	d	d	d
ST5066	Arzdorf	Germany	50°35′/10°50′	d	d	d	d	m	d	d	d
SK884	Behrensdorf	Germany	50°50′/10°60′	d	d	d	d	d	d	d	d
SK1986	Degerndorf	Germany	45°50′/10°25′	d	d	d	d	d	d	d	d
SU 639	Flieden	Germany	50°25′/10°35′	d	d	d	d	d	d	d	d
SK 882	Gröna	Germany	50°45′/10°45′	d	d	d	d	d	d	d	d
SK 898	Hamersen	Germany	55°15′/10°30′	d	d	d	d	d	d	d	d
SK1339	Kalitz	Germany	50°50′/10°10′	d	d	d	d	d	d	d	d
SK969	Kerchau	Germany	50°10′/10°10′	d	d	d	d	d	d	d	d
SK1803	Kübelhof	Germany	50°10′/10°25′	d	d	d	d	d	d	d	d
SK1330	Kümmernitz	Germany	50°50′/10°15′	d	d	d	d	d	d	d	d
SK 902	Lindtorf	Germany	50°41′/10°55′	d	d	d	d	m	d	d	d
SK 895	Lübz	Germany	55°30′/10°60′	d	d	d	m	d	d	d	d
SK 85	München	Germany	50°10′/10°30′	d	m	d	m	d	d	d	d
SK1335	Mützdorf	Germany	55°5′/10°15′	d	d	d	d	d	d	d	d
SK968	Rehfeld	Germany	50°55′/10°20′	d	d	d	d	d	d	d	d
SK635	Rückers	Germany	50°25′/10°35′	d	d	d	d	d	d	d	d
SK1062	Sandreuth	Germany	50°00′/11°35′	d	d	d	d	d	d	d	d
SU616	Schweben	Germany	50°25′/10°35′	d	d	d	d	d	d	d	d
SK1104	Straas	Germany	50°10′/10°45′	d	d	d	d	d	d	d	d
SK947	Suckow	Germany	55°25′/10°20′	d	d	d	d	d	d	d	d
SK942	Weitendorf	Germany	55°55′/10°20′	d	m	d	d	d	d	d	d
SK164	Migiondo	Italy	45°20′/10°20′	d	d	d	d	d	d	d	d
ST9598	Scar, Sanday Is.	UK	60°15′/-2°35′	d	d	d	d	d	d	d	d
ST9601	Scar, Sanday Is.	UK	60°15′/-2°35′	d	d	d	d	d	d	d	d
SK1683	Grosshain	Austria	48°15′/15°40′	m	m	m	m	m	m	m	m
SK1680	Pyhra	Austria	50°10′/15°40′	m	m	m	m	m	m	m	m
SK1	Silz	Austria	47°15′/10°55′	m	m	m	m	m	m	d	m
SK1790	Thallern	Austria	50°15′/15°35′	m	m	m	m	m	m	m	m
ST9569	Buškovice	Czech rep.	50°15′/12°20′	m	m	m	m	m	m	m	m
ZH27	Náměšt' n. O.	Czech rep.	50°10′/15°10′	m	m	m	m	m	m	m	m
SK1537	Náměšt' n. O.	Czech rep.	50°10′/15°10′	m	m	m	m	m	m	m	m
SK1783	Studenec	Czech rep.	50°10′/15°05′	m	m	m	m	m	m	m	m
SU1691	Žihle	Czech rep.	50°05′/15°25′	m	m	m	m	m	m	m	m
SK850	Blindow	Czech rep.	55°20′/15°55′	d	m	m	m	m	m	m	m
SK838	Grimme	Germany	55°25′/15°10′	m	m	m	m	m	m	m	m
SK1774	Gross Wokern	Germany	55°45′/10°30′	d	m	m	m	m	m	m	m
SK839	Hohenstein	Germany	50°35′/15°60′	m	m	m	m	m	m	m	m
SK974	Lalendorf	Germany	55°45′/10°30′	d	m	m	m	m	m	m	m
SK844	Lindhorst	Germany	55°25′/15°45′	d	m	m	m	m	d	m	m
ST8371	Debrecen	Hungary	45°30′/20°40′	m	m	m	m	m	m	m	m
ST8387	Gábortelep	Hungary	45°30′/20°55′	m	m	m	m	m	m	m	m
ST8304	Bielany	Poland	50°20′/20°15′	m	m	m	m	m	m	m	m
ST8324	Bozy Dar	Poland	50°00′/20°40′	m	m	m	m	m	m	m	m
SK859	Sliwnik	Poland	50°30′/15°30′	m	m	m	m	m	m	m	m
ST 8313	Zminne	Poland	50°40′/20°45′	m	m	m	m	m	m	m	m
ST8276	Cejkov	Slovakia	50°30′/20°45′	m	m	m	m	m	m	m	m
SK1672	Dubová	Slovakia	50°20′/15°20′	m	m	m	m	m	m	m	m
ST8225	Holčíkovce	Slovakia	50°00′/20°45′	m	m	m	m	m	m	m	m
ST8242	Nižný Hrušov	Slovakia	50°50′/20°45′	m	m	m	m	m	m	m	m
ST8257	Piesky	Slovakia	50°40′/20°50′	m	m	m	m	m	m	m	m
SK1669	Šenkvice	Slovakia	50°15′/15°20′	m	m	m	m	m	m	m	m
ST8272	Zempl. Jastrabie	Slovakia	50°30′/20°45′	m	m	m	m	m	m	m	m
IS7824	SCHEST	Germany	50°25′/10°35′	d	d	d	d	d	d	d	d
IS6960	SIT	UK	60°20′/-2°35′	d	d	d	d	d	d	d	d

Subspecific affiliations of the six remaining WDS were known *a priori* (see Material and Method).

Ten males of eight WDS representing Mmd (SCHEST, SIT, STRA, STRB) and Mmm (BUSNA, PWD/Ph, STUS, STUF) subspecies were studied. STRA, STRB and BUSNA strains were derived from wild populations 50 km east or west of the approximated hybrid zone center at their given latitudes [35]. STUS, STUF and PWD/Ph mice came from allopatric Mmm populations in the Czech Republic [35], [36]. All these strains were fully inbred (i.e. more than 20 generations of brother-sister mating) at the time of the study. The ancestors of the SCHEST strain were caught in Central Germany (Schweben, Hessen: [N: 50° 26′, E: 9° 35′]) in 2007. A parental pair of the SIT strain was sampled at Scar, Whitemill Bay, Sanday Island (Orkneys, Scotland [N: 59° 18′, E: -2° 33′]) in 2006. These two strains had undergone 4-10 and 4-9 generations of inbreeding, respectively, at the time of the study. Subspecific affiliations of the BUSNA, PWD, STRA, STRB, STUS and STUF strains were known *a priori* [35], [36]. The SCHEST and SIT mice were analyzed for the same eight diagnostic loci and were found to carry Mmd alleles (Table 1).

The animals were kept at the barrier-free facility of the Institute of Vertebrate Biology in Studenec and their care was in accordance with the standards set by the Czech Republic Act for Experimental Work, with animals fully compatible with corresponding regulations and standards of the European Union (license 227203/2011-MZE-17214). The experimental protocol was approved by the Committee on the Ethics of Animal Experiments of the Institute of Vertebrate Biology (Permit Number 27/2007). All mice were kept in polypropylene cages with bedding material under standard conditions: 20-22°C, 14/10-hr light/dark cycle. Tap-water and mouse pelleted food (St1, VELAZ, Prague, Czech Republic) were available *ad libitum*. Male mice were housed either individually or in pairs with a female of the same strain, and were sacrificed when age reached at least 60 days (Table 2). The paired males were separated for a minimum of five days to be kept in single cages preceding sacrifice and sperm collection.

**Table 2:** Mean values and respective standard errors (SE) of sperm component size for each subspecies and strain (scale in µm).

Genome	Mouse origin	N	Head length	Head width	Midpiece length	Tail length	Total length	*Day/Age range
Mmd	wild	28	8.05	3.29	21.43	94.86	124.34	1-267
(SE)			0.05	0.02	0.09	0.37	0.30	
SCHEST	strain	10	8.20	3.41	20.22	97.92	126.35	127-288
(SE)			0.08	0.03	0.15	0.62	0.67	
STRA	strain	10	8.40	3.53	21.34	97.89	127.63	65-399
(SE)			0.08	0.03	0.15	0.62	0.67	
STRB	strain	10	7.95	3.16	21.24	96.75	125.95	62-286
(SE)			0.08	0.03	0.15	0.62	0.67	
SIT	strain	10	8.27	3.21	21.80	98.33	128.41	100-259
(SE)			0.08	0.03	0.15	0.62	0.67	
Mmm	wild	28	8.53	3.37	22.58	95.18	126.35	1-275
(SE)			0.05	0.02	0.09	0.37	0.40	
BUSNA	strain	10	8.88	3.54	21.93	96.30	127.11	60-409
(SE)			0.08	0.03	0.15	0.62	0.67	
PWD	strain	10	8.72	3.46	23.02	96.67	128.40	60-310
(SE)			0.08	0.03	0.15	0.62	0.67	
STUF	strain	10	8.03	3.44	22.87	94.07	124.97	63-375
(SE)			0.08	0.03	0.15	0.62	0.67	
STUS	strain	10	8.31	3.36	22.09	96.86	127.27	100-290
(SE)			0.08	0.03	0.15	0.62	0.67	

*In wild mice, age ranges are replaced by number of days spent in captivity.

The study protocol did not allow for absolute age to be completely controlled. The absolute age of caught wild mice was unknown. Additionally, individuals were kept in captivity for varying numbers of days before sacrifice (Table 2). We tried to control for the second limitation by choosing wild males for comparison that had, on average (mean 179 days, F_7,72_  = 0.32, P = 0.94), spent the same amount of time in captivity. Contrary to wild mice, the age of WDS males was known. Two criteria for choosing experimental males were applied: only males aged more than 60 days were scored, and males were selected to have, on average, the same age between the Mmd and Mmm groups. After dislocation, left cauda epididymides were dissected and transferred into the medium (DMEM, Invitrogen, Germany), heated to 37°C, and punctured by needles that released sperm into the medium. Sperm were allowed to swim freely for five minutes. A drop of sperm suspension was smeared on a slide, air-dried, and fixed in 5% Giemsa solution (Sigma-Aldrich, Germany) for 15 min.

**Figure 1:**
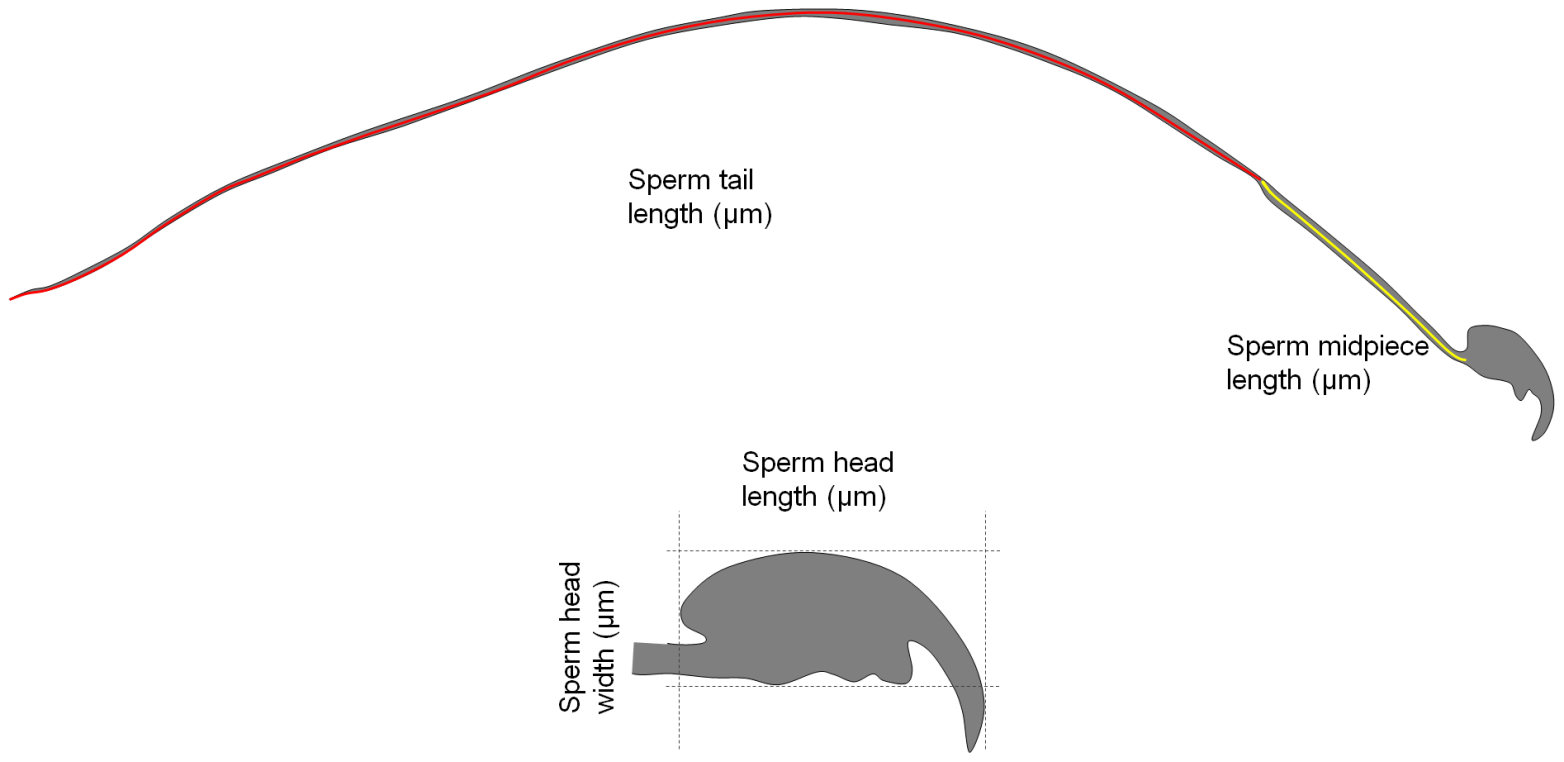
Schema of mouse sperm component measurements. Measures of sperm midpiece length and tail length are highlighted in yellow and red. Sperm head length and width measurements are represented by dashed lines.

Images of sperm were captured via microscopy (BX51, Olympus, Czech Republic) and digital camera (DP71, Olympus) at 400× magnification. Four sperm dimensions: head length, head width, midpiece length, and tail length (Fig. 1) were measured with Olympus imaging software (QuickPHOTO Industrial 2.3, Olympus). Ten sperm were measured per individual. The measurement accuracy was to one tenth of a micrometer (µm). For statistical analysis, one-way ANOVA on sample means was performed. We used the dataset for three separate tests (wild Mmd vs. wild Mmm; wild vs. individual WDS; wild vs. subspecies-specific WDS grouped together). Consequently, Bonferroni adjustments were applied to correct for multiple comparisons and omnibus test type I error was set to α = 0.05/3. When significant differences between groups were detected, Tukey post-hoc contrast analysis was employed to specify among-group differences.

To quantify the reliability of WDS as a surrogate for sperm size variation in wild mice, linear discriminant analysis was applied as implemented in the MASS package of R environment (http://cran.r-project.org). Cross-validated discriminant scores were calculated for significantly different sperm size variables (sperm head length, sperm head width, and midpiece length) separately for wild and WDS mice. Success of classification of mice into Mmd or Mmm as predicted by discriminant function was compared to subspecific origin as assessed by genetic analysis.

## Results

Molecular analyses of six diagnostic markers separated wild mice into two genetically distinct groups (Table 1). Genotypes were mostly in accordance with expected geographic position of Mmd and Mmm subspecies [16], [17]. One exception was an individual mouse from a locality in Western Austria (Silz: 47°15′ N/10°55′ E) that carried Mmm alleles. Consequently, this individual was treated as Mmm in further analyses.

**Figure 2:**
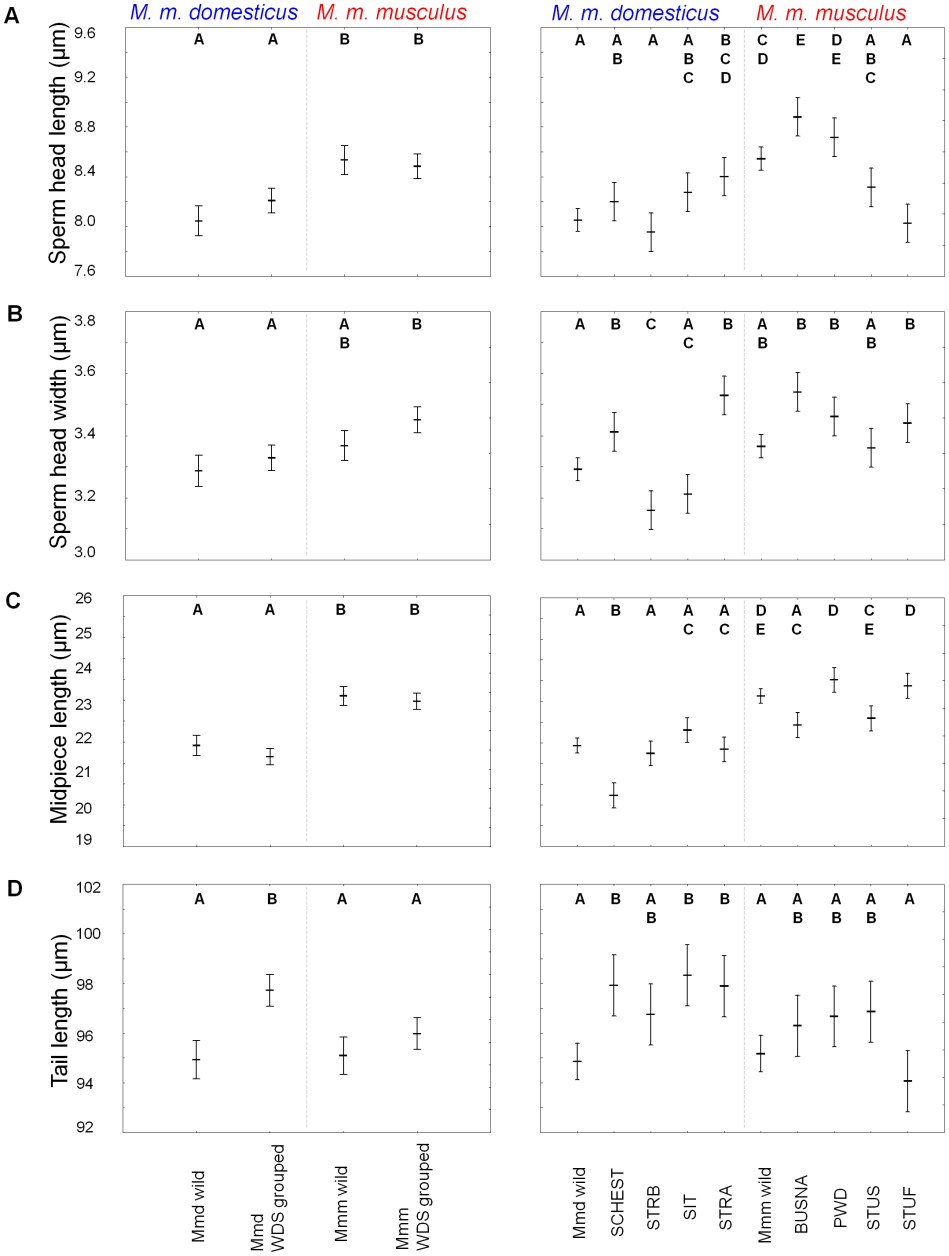
Differences between Mmd and Mmm in sperm component size. Sperm head length (A), sperm head width (B), midpiece length (C), and tail length (D). Individual wild-derived strains are marked separately on the left side of the graphs while subspecies-specific strains are put together on the right side of the graphs. The same upper-case letters indicate similarity of sperm components between subspecies and strains in the Tukey *post hoc* test comparison. Error bars represent 95% confidence intervals.

The sperm component measurements of wild individuals are available in S1 Table. The wild Mmd and Mmm groups differed in terms of midpiece length (F_1,54_ = 48.54, P<0.001), sperm head length (F_1,54_ = 38.15, P<0.001), and head width (F_1,54_ = 6.52, P = 0.01); in all cases, values were higher in Mmm as compared to Mmd (Fig. 2, Table 2). Given that Mmd and Mmm differed in two sperm length components, it is not surprising that these two groups also differed in total sperm length (F_1,54_ = 6.67, P = 0.01). The only non-significant variable reported was sperm tail length (F_1,54_ = 0.21, P = 0.65).

**Figure 3:**
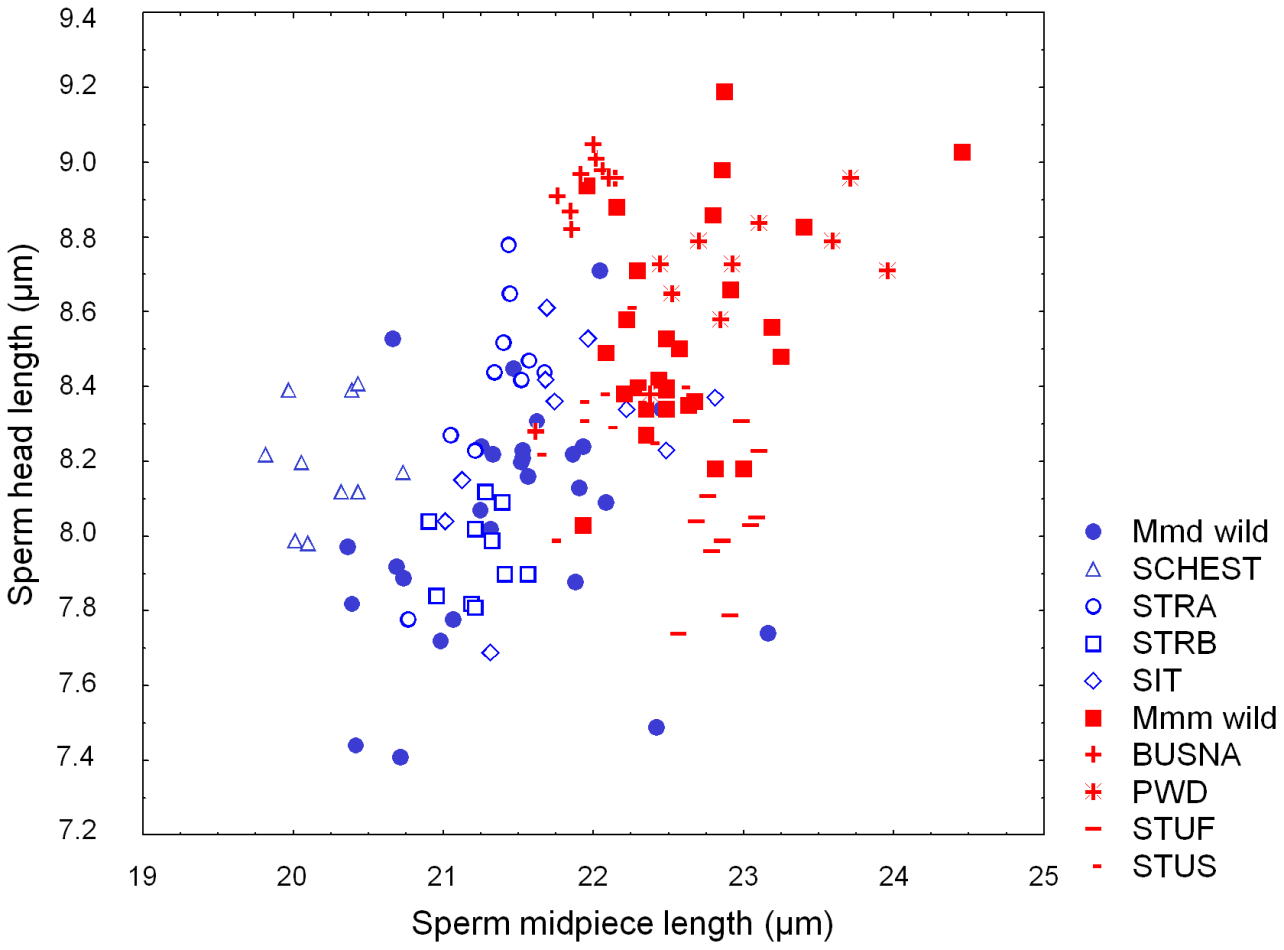
Scatter-plot of sperm head length and midpiece length for all individual males analyzed. Mmd-specific mice are shown in blue and Mmm-specific mice in red.

A scatterplot of wild mice in a reduced morphospace defined by the two most statistically significant variables revealed only marginal mixing of the two groups (1 Mmm within Mmd and 2 Mmd within Mmm in Fig. 3). Discriminant analysis based on three significantly different variables (sperm head length, sperm head width, and midpiece length) assigned 52 out of 56 (92.9%) wild mice correctly to their respective genetic backgrounds (Table 3).

**Table 3:** Discriminant analysis of sperm size components calculated separately for wild mice and both wild and inbred mice.

	*M. m. domesticus*		*M. m. musculus*		Percent correct	
	wild	all	wild	all	wild	all
*M. m. domesticus*	25	57	3	11	89.3	83.8
*M. m. musculus*	1	5	27	63	96.4	92.6

Number of mice in lines was obtained via genetic analysis; mice in columns were assigned by discriminant analysis.

The subspecies-attributed sperm size differentiation showed a similar pattern when WDS mice that were grouped according to subspecies were added to morphometric analysis (Fig. 2). Tukey post-hoc contrast clearly separated Mmm and Mmd mice at midpiece length (F_3,132_ = 46.50, P<0.001) and sperm head length (F_3,132_ = 16.58, P<0.001). The subspecific partitioning of phenotypic variability was less clear in the remaining variables. Although the four groups differed in terms of sperm head width (F_3,132_ = 9.82, P<0.001), only Mmm-WDS had significantly wider sperm heads (Fig. 2, S2 Table). Similarly, differentiation was observed in terms of sperm tail length (F_3,132_ = 13.85, P<0.001), but this could be attributed to Mmd-WDS having longer tails, on average (Fig. 2, S2 Table). The differences were also found in the total sperm length (F_3,132_ = 9.77, P<0.001). Tukey post-hoc contrast separated wild Mmd possessing shorter sperm from the other three groups of mice (mean±standard deviation, 124.26±1.70 µm for wild Mmd compared to 126.82±2.01 µm representing average for wild and Mmm-WDS and Mmd-WDS). Discriminant analysis utilizing the same three length measures as mentioned in the previous paragraph performed slightly worse when all mice were analyzed. The summed value of correct assignments of wild and inbred mice was 120/136 (88.2%) with respect to their Mmd and Mmm genotypes (Table 3).

When individual strains were compared separately, variation in most traits could be partitioned into more detailed differentiation. Significant differentiation was found in all sperm variables (maximum F_9,126_ = 36.33 for midpiece, minimum F_9,126_ = 6.83 for total sperm length; P<0.001 for all traits). While the post-hoc tests of mice grouped over subspecies divided variation into two levels mostly corresponding to Mmd and Mmm, up to five different levels were discerned by Tukey post-hoc contrast analysis (cf. left and right panels in Fig. 2; all pairwise comparisons are listed in S2 Table). Individual WDS did differ in terms of sperm size not only between, but also within the same subspecies. In some WDS, sperm size components moved from subspecies-specific values. This pattern can be exemplified by the STUF (Mmm) males who have sperm head lengths similar to that of the average Mmd mouse (Figs. 2 and 3, Table 2). The direction of the intersubspecific shifts was not always predictable for all sperm traits. For example, SCHEST (Mmd) males had midpiece lengths lower than wild Mmd but were indistinguishable from any group of Mmm males in terms of sperm head width (Fig. 2).

## Discussion

An important finding of this study was that Mmm and Mmd house mouse subspecies substantially differed in terms of certain sperm size components. Despite the presence of geographic variation in sperm size documented in wild mice (Table 1, Fig. 3), spermatozoa of wild Mmm were characterized by significantly larger head and midpiece length than spermatozoa of Mmd (Fig. 2).

Breeding conditions of all individuals were standardized, and therefore cannot be responsible for the observed variation in sperm traits. Similarly, although males were sacrificed at different ages, or days spent in captivity (see Table 2 for ranges), we controlled for this effect by choosing WDS males that have the same age when processed. Additionally, previous research has demonstrated that there are no age-specific effects on head area and midpiece length in laboratory mouse spermatozoa [37].

Although there is a strong phylogenetic component that influences sperm morphology [9], [38], it is unlikely to be the primary explanatory factor for the differences seen in this study. The two house mouse subspecies started to diverge roughly 350,000 years ago [14], currently forming a monophyletic clade within the genus *Mus* [39], [40]. To explain factors shaping sperm size evolution, one would have to further explore differences in mating systems and sexual selection within these mouse lineages. Promiscuity and male-male sperm competition in female's reproductive tract is a result of post-copulatory sexual selection [9], [41], which causes variance in morphology of sperm [38]. Sperm morphology influences other sperm traits such as sperm velocity (e.g. in primates [42], birds [12], and mice [26]), and is ultimately one of the main indicators of a male's success when sperm competition occurs [2], [3]. However, some controversy holds on this subject as no such associations were reported to be likely in red deer or in mice [20], [43].

Promiscuity has evolved in both subspecies. Multiple paternity was reported to range between 23-30% in Mmd [28], [29], [44] and 29% in Mmm [45], and is not sufficient to explain the differences observed in sperm size between the mouse subspecies. Previous studies have reported that Mmm and Mmd subspecies differ in terms of sperm velocity, with velocity being higher in Mmm than Mmd [20], [46]. Analysis of 180 males sampled directly from the HMHZ, however, did not reveal significant differences in sperm motility between the subspecies [19]. Wild males of Mmm were shown to display lower sperm counts than their Mmd counterparts [19]. The larger sperm size can indicate a trade-off between sperm number and size in different taxa, e.g. [47]. However, there is no evidence to suggest trade-offs between sperm traits in muroid species [48].

Studies inferring selection and genetic basis of sperm size differences may benefit from including natural variation found regularly in wild animal specimens. One focus of the current study was to assess the reliability of wild-derived strains as proxies for processes occurring in the wild. Analysis of within-subspecies groups of WDS and their wild conspecifics showed consistent patterns in two morphometric variables (cf. sperm head and midpiece lengths in Fig. 2). This finding is promising as it gives support to WDS as a suitable model for studies on sperm size evolution of the house mouse. However, closer inspection has revealed differences in sperm morphology between particular strains of both Mmd and Mmm. For example, STUF (Mmm) spermatozoa have shorter heads than other Mmm individuals. Under *post hoc* comparison, these spermatozoa are clustered with wild Mmd and three Mmd-WDS: SCHEST, STRB, and SIT (Fig. 2, Table 2). Similarly, the STRA (Mmd) males were outliers within Mmd, having sperm head width similar to Mmm mice (Fig. 2, Table 2). In any case, the next logical step should be to determine which factors cause these strain-specific differences.

The most obvious explanation for strain-specific differences in sperm dimension lies in the very process of WDS derivation. Usually, a single (randomly chosen) individual can be selected as progenitor of the strain, and may inevitably preserve only limited genetic variation of a natural population. Given that there is a heritable component of sperm design [23], [49]-[51], sons will most likely display sperm traits similar to their father. Consequently, individual WDS mice will only present a snap-shot of phenotypic variation depending on the values of the progenitor pair selected to form the WDS. This possibility is exemplified in Fig. 3, which shows phenotypic variation in sperm head length and midpiece length. Although both variables are significantly larger in Mmm than in Mmd (Fig. 2, Table 2), high phenotypic variation was detected at subspecific levels. Values of wild mice and WDS from both subspecies were partly overlapping. The most probable explanation for the distribution of STUF, STUS and SIT males within the morphospace seems to be due to a lineage stemming from ancestral parents with extraordinary phenotypes. Alternative explanations may lie in mouse divergence and/or colonization history. House mouse genomes have a large degree of incomplete lineage sorting [52]-[54]. Therefore, these phenotypes could reflect ancestral polymorphisms for sperm traits still segregating among the subspecies. Additional sampling will be necessary among wild-caught subspecies to determine how extraordinary these phenotypes are.

Strain-specific sperm dimensions may additionally be viewed from a second perspective. WDS with extreme sperm component values are potentially suitable sources for quantitative trait loci (QTL) mapping from F2 or a backcrossed offspring. Distribution of individual mice in Fig. 3 reveals the presence of a paired WDS lying at the opposite edge of morphospace. Consequently, both intrasubspecific crosses (i.e. crosses that would avoid negative interaction between incompatible loci, such as BUSNA x STUF in Mmm) or intersubspecific crosses (i.e. those seeing for effects of hybridization like PWD x STRB) may be implemented to map QTL of sperm size.

The genetic analysis of wild mice broadens our understanding of the two house mouse subspecies borders. The finding of an individual that carries both mitochondrial and sex-linked traits of Mmm genome in a region where the presence of Mmd mice was expected [17] is rather surprising. Nevertheless, these data are in agreement with a recent report of a mouse from a neighboring village in Tyrol (Staudach: 47°16′ N/10°57′ E; 2.5 km apart from previous locality) that carries mtDNA of Mmm origin [55]. These findings of two independent Mmm samples far westward of the house mouse hybrid zone in the Alpine valley of the Inn River may indicate the presence of another, undescribed stretch of subspecies contact. However, we cannot exclude the possibility that this unexpected finding of two Mmm samples is the result of long-distance human-mediated migration.

## Supporting Information

S1 Table **Mean values of sperm dimensions (in µm) per sperm sample and standard deviation (±SD) of wild house mice.**
(PDF)

S2 Table **Post-hoc comparisons of differences in selected sperm traits between wild Mmd (MmdW), wild Mmm (MmmW) and wild-derived strains (WDS).** Shown are results of Tukey post-hoc tests for comparisons of MmdW, MmmW and either WDS grouped (MmdWDS for *M. m. domesticus* strains and MmmWDS for *M. m. musculus* strains, panels A-E) or WDS treated separately (Mmm-WDS: BUSNA, PWD, STUS, STUF; Mmd-WDS: SCHEST, STRB, SIT, STRA, panels F-J). (P_adj_ - P value adjusted for multiple comparisons).
(PDF)
