# Correction: Sperm Morphology in Two House Mouse Subspecies: Do Wild-Derived Strains and Wild Mice Tell the Same Story?

**DOI:** 10.1371/journal.pone.0120484

**Published:** 2015-03-12

**Authors:** The PLOS ONE Staff

There are errors in the Funding section. The correct funding information is as follows: Research was supported by the Czech Science Foundation (206–08–0640), the Ministry of Education, Youth and Sport of the Czech Republic (SVV260 087/2014), and the National Science Foundation (DEB0746560). Mouse pellets and genotyping of allopatric mice were reimbursed from the Czech Science Foundation (P506–11–1792). The funders had no role in study design, data collection, data analysis, decision to publish, or preparation of the manuscript.
