# Supplementary material for: Sperm Morphology in Two House Mouse Subspecies: Do Wild-Derived Strains and Wild Mice Tell the Same Story?
Source: PLoS One. 2014 Dec 26;9(12):e115669. doi: 10.1371/journal.pone.0115669 (PMC4277342; doi:10.1371/journal.pone.0115669)
Supplement: S1 Table — Mean values of sperm dimensions (in µm) per sperm sample and standard deviation (±SD) of wild house mice. (PDF) [file pone.0115669.s001.pdf]

Table S1 Mean values of sperm dimensions (in µm) per sperm sample and standard deviation (±SD) of wild house mice.

Genome	Locality	Country	Sperm components size (µm) (±SD)				
			Head length	Head width	Midpiece	Tail	Total sperm
Mmd	Feldkirch	Austria	7.44±0.21	3.29±0.10	20.41±0.26	92.98±2.55	120.83±2.47
Mmd	Sinj	Croatia	8.22±0.21	3.15±0.10	21.33±0.31	93.22±1.43	122.77±1.50
Mmd	Brouzet-lès-Quissac	France	8.24±0.25	3.32±0.12	21.93±0.46	98.81±1.24	128.98±1.11
Mmd	St-Jean-et-Royans	France	8.07±0.30	3.30±0.08	21.24±0.44	96.70±2.15	126.01±2.38
Mmd	Arzdorf	Germany	7.92±0.27	3.45±0.16	20.69±0.33	95.23±1.58	123.84±1.73
Mmd	Behrensdorf	Germany	7.82±0.16	3.26±0.13	20.39±0.44	94.84±1.61	123.05±1.78
Mmd	Degerndorf	Germany	7.49±0.18	3.03±0.12	22.42±1.06	89.64±2.83	119.55±2.94
Mmd	Flieden	Germany	8.02±0.30	3.36±0.08	21.31±0.34	97.49±5.42	126.82±5.34
Mmd	Gröna	Germany	7.88±0.28	3.34±0.07	21.88±0.15	96.15±1.30	125.91±1.33
Mmd	Hamersen	Germany	8.23±0.35	3.31±0.06	21.53±0.58	94.18±1.19	123.94±1.16
Mmd	Kalitz	Germany	7.78±0.32	3.13±0.05	21.06±0.32	94.40±1.05	123.24±1.14
Mmd	Kerchau	Germany	8.31±0.32	3.34±0.11	21.62±0.88	92.07±1.85	122.00±1.61
Mmd	Kübelhof	Germany	7.74±0.24	3.22±0.12	23.16±0.66	89.79±1.81	120.69±1.49
Mmd	Kümmernitz	Germany	8.09±0.22	3.29±0.07	22.08±0.63	95.85±2.24	126.02±1.76
Mmd	Lindtorf	Germany	8.34±0.41	3.36±0.08	22.45±0.63	96.57±2.74	127.36±3.09
Mmd	Lübz	Germany	8.21±0.24	3.40±0.05	21.53±0.40	96.45±1.06	126.15±1.22
Mmd	München	Germany	8.16±0.25	3.32±0.11	21.56±0.62	95.32±1.35	125.04±1.17
Mmd	Mützdorf	Germany	7.41±0.24	3.19±0.10	20.71±0.40	89.07±2.60	117.19±2.59

Mmd	Rehfeld	Germany	8.22±0.38	3.42±0.10	21.86±0.70	92.91±2.16	122.99±2.05
Mmd	Rückers	Germany	8.24±0.27	3.28±0.10	21.25±0.34	98.55±1.71	128.04±1.75
Mmd	Sandreuth	Gemany	8.20±0.33	3.37±0.13	21.52±0.39	94.62±2.39	124.34±2.70
Mmd	Schweben	Germany	7.97±0.21	3.23±0.12	20.36±0.32	96.01±1.11	124.34±1.06
Mmd	Straas	Germany	8.71±0.22	3.41±0.09	22.04±0.82	93.6±2.42	124.35±2.19
Mmd	Suckow	Gemany	8.13±0.29	3.19±0.09	21.90±0.20	91.38±1.34	121.41±1.40
Mmd	Weitendorf	Germany	8.53±0.26	3.43±0.11	20.66±0.42	94.95±1.95	124.14±2.06
Mmd	Migiondo	Italy	7.89±0.24	3.30±0.05	20.73±0.20	99.15±1.18	127.77±1.01
Mmd	Scar, Sanday Is.	UK	7.72±0.36	3.18±0.08	20.98±0.58	94.58±1.54	123.28±1.67
Mmd	Scar, Sanday Is.	UK	8.45±0.16	3.29±0.09	21.47±0.22	101.47±1.17	131.39±1.15
Mmm	Grosshain	Austria	8.27±0.26	3.39±0.14	22.35±0.41	88.93±1.34	119.55±1.26
Mmm	Pyhra	Austria	8.56±0.17	3.44±0.08	23.19±0.45	95.34±1.03	127.09±1.23
Mmm	Silz	Austria	8.40±0.29	3.66±0.11	22.48±0.35	96.57±1.61	127.45±1.48
Mmm	Thallern	Austria	8.18±0.12	3.37±0.14	22.81±1.37	95.57±2.83	126.56±3.34
Mmm	Buškovice	Czech Rep.	8.39±0.26	3.31±0.09	22.48±0.33	98.24±1.90	129.11±1.85
Mmm	Čikov	Czech Rep.	8.94±0.18	3.53±0.08	21.95±0.28	95.11±2.52	126.00±2.53
Mmm	Náměšť n. O.	Czech Rep.	8.03±0.14	3.17±0.05	21.93±0.66	94.71±0.65	124.67±1.05
Mmm	Studenec	Czech Rep.	8.40±0.31	3.55±0.08	22.30±0.37	95.95±2.39	126.65±2.40
Mmm	Žihle	Czech Rep.	8.49±0.20	3.25±0.11	22.08±0.37	96.26±1.21	126.83±1.12
Mmm	Blindow	Germany	8.35±0.29	3.41±0.11	22.63±0.53	95.56±2.42	126.54±2.71
Mmm	Grimme	Germany	8.86±0.27	3.48±0.09	22.79±0.46	98.21±1.22	129.86±1.08
Mmm	Gross Wokern	Germany	8.58±0.33	3.25±0.12	22.22±0.46	96.73±2.21	127.53±2.02

Mmm	Hohenstein	Germany	8.71±0.17	3.46±0.13	22.29±0.40	93.40±1.25	124.40±1.35
Mmm	Lalendorf	Germany	8.18±0.33	3.40±0.12	23.00±0.51	97.61±4.76	128.79±4.78
Mmm	Lindhorst	Germany	8.50±0.36	3.20±0.12	22.57±0.58	93.36±0.94	124.43±0.86
Mmm	Debrecen	Hungary	8.66±0.31	3.46±0.05	22.91±0.31	93.9±1.46	125.47±1.75
Mmm	Gábortelep	Hungary	9.03±0.20	3.60±0.12	24.45±0.52	99.90±1.87	133.38±2.06
Mmm	Bielany	Poland	8.38±0.23	3.28±0.18	22.20±0.37	94.36±3.74	124.94±3.71
Mmm	Bozy Dar	Poland	8.53±0.33	3.14±0.13	22.48±0.43	90.83±1.33	121.84±1.27
Mmm	Sliwnik	Poland	8.88±0.35	3.49±0.10	22.15±0.60	94.46±3.39	125.49±3.78
Mmm	Zminne	Poland	8.98±0.30	3.44±0.07	22.85±0.39	92.73±2.02	124.56±1.90
Mmm	Cejkov	Slovakia	8.83±0.24	3.39±0.12	23.40±0.95	96.90±1.20	129.13±1.82
Mmm	Dubová	Slovakia	8.34±0.25	3.28±0.16	22.48±0.37	95.09±1.44	125.91±1.89
Mmm	Holčíkovce	Slovakia	9.19±0.25	3.14±0.12	22.87±0.79	97.67±0.90	129.73±1.18
Mmm	Nižný Hrušov	Slovakia	8.42±0.37	3.45±0.12	22.43±0.22	93.7±1.51	124.55±1.36
Mmm	Piesky	Slovakia	8.48±0.26	3.31±0.09	23.25±1.10	94.99±1.98	126.72±1.32
Mmm	Šenkvice	Slovakia	8.36±0.46	3.19±0.10	22.67±0.82	92.25±1.64	123.28±2.06
Mmm	Zempl. Jastrabie	Slovakia	8.34±0.25	3.21±0.10	22.35±0.18	96.52±1.29	127.21±1.27
