# Supplementary material for: Sperm Morphology in Two House Mouse Subspecies: Do Wild-Derived Strains and Wild Mice Tell the Same Story?
Source: PLoS One. 2014 Dec 26;9(12):e115669. doi: 10.1371/journal.pone.0115669 (PMC4277342; doi:10.1371/journal.pone.0115669)
Supplement: S2 Table — Post-hoc comparisons of differences in selected sperm traits between wild Mmd (MmdW), wild Mmm (MmmW) and wild-derived strains (WDS). Shown are results of Tukey post-hoc tests for comparisons of MmdW, MmmW and either WDS grouped (MmdWDS for M. m. domesticus strains and MmmWDS for M. m. musculus strains, panels A-E) or WDS treated separately (Mmm-WDS: BUSNA, PWD, STUS, STUF; Mmd-WDS: SCHEST, STRB, SIT, STRA, panels F-J). (Padj - P value adjusted for multiple comparisons). (PDF) [file pone.0115669.s002.pdf]

Table S2 Post-hoc comparisons of differences in selected sperm traits between wild Mmd (MmdW), wild Mmm (MmmW) and wild-derived strains (WDS). Shown are results of Tukey post hoc tests for comparisons of MmdW, MmmW and either WDS grouped (MmdWDS for *M. m. domesticus* strains and MmmWDS for *M. m. musculus* strains, panels A-E) or WDS treated separately (Mmm-WDS: BUSNA, PWD, STUS, STUF; Mmd-WDS: SCHEST, STRB, SIT, STRA, panels F-J). (P_{adj} - P value adjusted for multiple comparisons).

A. Sperm head length

Comparison	Difference in means	Lower Cl95%	Upper Cl95%	P_{adj}
MmdW-MmdWDS	-0.155	-0.357	0.046	0.189
MmmWDS-MmdWDS	0.278	0.095	0.460	< 0.001
MmmW-MmdWDS	0.327	0.126	0.528	< 0.001
MmmWDS-MmdW	0.433	0.232	0.634	< 0.001
MmmW-MmdW	0.483	0.2645	0.701	< 0.001
MmmW-MmmWDS	0.050	-0.152	0.251	0.918

B. Sperm head width

Comparison	Difference in means	Lower Cl95%	Upper Cl95%	P_{adj}
MmdW-MmdWDS	-0.037	-0.121	0.047	0.660
MmmWDS-MmdWDS	0.123	0.047	0.199	< 0.001
MmmW-MmdWDS	0.044	-0.040	0.128	0.531
MmmWDS-MmdW	0.160	0.076	0.243	< 0.001
MmmW-MmdW	0.081	-0.010	0.171	0.101
MmmW-MmmWDS	-0.079	-0.163	0.005	0.072

C. Sperm midpiece length

Comparison	Difference in means	Lower Cl95%	Upper Cl95%	P_{adj}
MmdW-MmdWDS	0.278	-0.124	0.681	0.279
MmmWDS-MmdWDS	1.324	0.959	1.689	< 0.001
MmmW-MmdWDS	1.432	1.030	1.835	< 0.001
MmmWDS-MmdW	1.046	0.644	1.449	< 0.001
MmmW-MmdW	1.154	0.718	1.591	< 0.001
MmmW-MmmWDS	0.108	-0.294	0.511	0.897

D. Sperm tail length

Comparison	Difference in means	Lower Cl95%	Upper Cl95%	P_{adj}
MmdW-MmdWDS	-2.867	-4.176	-1.558	< 0.001
MmmWDS-MmdWDS	-1.748	-2.936	-0.560	< 0.001
MmmW-MmdWDS	-2.546	-3.855	-1.237	< 0.001
MmmWDS-MmdW	1.119	-0.190	2.428	0.122
MmmW-MmdW	0.321	-1.099	1.740	0.936
MmmW-MmmWDS	-0.798	-2.107	0.511	0.389

E. Total sperm length

Comparison	Difference in means	Lower Cl95%	Upper Cl95%	P_{adj}
MmdW-MmdWDS	-2.744	-4.187	-1.301	< 0.001
MmmWDS-MmdWDS	-0.146	-1.455	1.163	0.991
MmmW-MmdWDS	-0.787	-2.229	0.656	0.490
MmmWDS-MmdW	2.598	1.155	4.041	< 0.001
MmmW-MmdW	1.958	0.394	3.522	0.008
MmmW-MmmWDS	-0.641	-2.083	0.802	0.656

F. Sperm head length

Comparison	Difference in means	Lower Cl95%	Upper Cl95%	P_{adj}
BUSNA-MmdW	0.830	0.542	1.118	< 0.001
PWD-MmdW	0.665	0.377	0.953	< 0.001
STUF-MmdW	-0.026	-0.314	0.262	1.000
STUS-MmdW	0.263	-0.025	0.551	0.104
MmmW-MmdW	0.483	0.274	0.691	< 0.001
SCHEST-MmdW	0.148	-0.140	0.436	0.817
SIT-MmdW	0.223	-0.065	0.511	0.281
STRA-MmdW	0.349	0.061	0.637	0.006
STRB-MmdW	-0.098	-0.386	0.190	0.984
PWD-BUSNA	-0.165	-0.514	0.184	0.881
STUF-BUSNA	-0.856	-1.205	-0.507	< 0.001
STUS-BUSNA	-0.567	-0.916	-0.218	< 0.001
MmmW-BUSNA	-0.347	-0.635	-0.060	0.006
SCHEST-BUSNA	-0.682	-1.031	-0.333	< 0.001
SIT-BUSNA	-0.607	-0.956	-0.258	< 0.001
STRA-BUSNA	-0.481	-0.830	-0.132	< 0.001
STRB-BUSNA	-0.928	-1.277	-0.579	< 0.001
STUF-PWD	-0.691	-1.040	-0.342	< 0.001
STUS-PWD	-0.402	-0.751	-0.053	0.011
MmmW-PWD	-0.182	-0.470	0.105	0.571
SCHEST-PWD	-0.517	-0.866	-0.168	< 0.001
SIT-PWD	-0.442	-0.791	-0.093	0.003
STRA-PWD	-0.316	-0.665	0.033	0.112
STRB-PWD	-0.763	-1.112	-0.414	< 0.001
STUS-STUF	0.289	-0.060	0.638	0.199
MmmW-STUF	0.509	0.221	0.796	< 0.001
SCHEST-STUF	0.174	-0.175	0.523	0.843
SIT-STUF	0.249	-0.100	0.598	0.398
STRA-STUF	0.375	0.026	0.724	0.025
STRB-STUF	-0.072	-0.421	0.277	1.000
MmmW-STUS	0.220	-0.068	0.507	0.301
SCHEST-STUS	-0.115	-0.464	0.234	0.987
SIT-STUS	-0.040	-0.389	0.309	1.000
STRA-STUS	0.086	-0.263	0.435	1.000
STRB-STUS	-0.361	-0.710	-0.011	0.037
SCHESTST-MmmW	-0.335	-0.622	-0.047	0.010
SIT-MmmW	-0.260	-0.547	0.028	0.115
STRA-MmmW	-0.134	-0.421	0.154	0.891
STRB-MmmW	-0.581	-0.868	-0.293	< 0.001
SIT-SCHEST	0.075	-0.274	0.424	1.000
STRA-SCHEST	0.201	-0.148	0.550	0.699
STRB-SCHEST	-0.246	-0.595	0.103	0.416
STRA-SIT	0.126	-0.223	0.475	0.977
STRB-SIT	-0.321	-0.670	0.028	0.100
STRB-STRA	-0.447	-0.796	-0.098	0.003

G. Sperm head width

Comparison	Difference in means	Lower Cl95%	Upper Cl95%	P_{adj}
BUSNA-MmdW	0.250	0.134	0.365	< 0.001
PWD-MmdW	0.171	0.055	0.286	< 0.001
STUF-MmdW	0.150	0.034	0.265	0.002
STUS-MmdW	0.070	-0.046	0.185	0.646
MmmW-MmdW	0.081	-0.003	0.165	0.071
SCHEST-MmdW	0.121	0.005	0.236	0.034
SIT-MmdW	-0.079	-0.195	0.036	0.457
STRA-MmdW	0.239	0.123	0.354	< 0.001
STRB-MmdW	-0.131	-0.247	-0.016	0.013
PWD-BUSNA	-0.079	-0.220	0.062	0.728
STUF-BUSNA	-0.100	-0.241	0.041	0.403
STUS-BUSNA	-0.180	-0.321	-0.039	0.003
MmmW-BUSNA	-0.169	-0.285	-0.053	< 0.001
SCHESTST-BUSNA	-0.129	-0.270	0.012	0.102
SIT-BUSNA	-0.329	-0.470	-0.188	< 0.001
STRA-BUSNA	-0.011	-0.152	0.130	1.000
STRB-BUSNA	-0.381	-0.522	-0.240	< 0.001
STUF-PWD	-0.021	-0.162	0.120	1.000
STUS-PWD	-0.101	-0.242	0.040	0.388
MmmW-PWD	-0.090	-0.206	0.026	0.280
SCHEST-PWD	-0.050	-0.191	0.091	0.979
SIT-PWD	-0.250	-0.391	-0.109	< 0.001
STRA-PWD	0.068	-0.073	0.209	0.866
STRB-PWD	-0.302	-0.443	-0.161	< 0.001
STUS-STUF	-0.080	-0.221	0.061	0.714
MmmW-STUF	-0.069	-0.185	0.047	0.659
SCHEST-STUF	-0.029	-0.170	0.112	1.000
SIT-STUF	-0.229	-0.370	-0.088	< 0.001
STRA-STUF	0.089	-0.052	0.230	0.574
STRB-STUF	-0.281	-0.422	-0.140	< 0.001
MmmW-STUS	0.011	-0.105	0.127	1.000
SCHEST-STUS	0.051	-0.090	0.192	0.976
SIT-STUS	-0.149	-0.290	-0.008	0.029
STRA-STUS	0.169	0.028	0.310	0.006
STRB-STUS	-0.201	-0.342	-0.060	< 0.001
SCHEST-MmmW	0.040	-0.076	0.156	0.983
SIT-MmmW	-0.160	-0.276	-0.044	< 0.001
STRA-MmmW	0.158	0.042	0.274	< 0.001
STRB-MmmW	-0.212	-0.328	-0.096	< 0.001
SIT-SCHEST	-0.200	-0.341	-0.059	< 0.001
STRA-SCHEST	0.118	-0.023	0.259	0.184
STRB-SCHEST	-0.252	-0.393	-0.111	< 0.001
STRA-SIT	0.318	0.177	0.459	< 0.001
STRB-SIT	-0.052	-0.193	0.089	0.973
STRB-STRA	-0.370	-0.511	-0.229	< 0.001

H. Sperm midpiece length

Comparison	Difference in means	Lower Cl95%	Upper Cl95%	P_{adj}
BUSNA-MmdW	0.498	-0.078	1.075	0.153
PWD-MmdW	1.585	1.009	2.162	< 0.001
STUF-MmdW	1.443	0.867	2.020	< 0.001
STUS-MmdW	0.657	0.081	1.234	0.013
MmmW-MmdW	1.154	0.736	1.573	< 0.001
SCHEST-MmdW	-1.206	-1.782	-0.629	< 0.001
SIT-MmdW	0.372	-0.204	0.949	0.545
STRA-MmdW	-0.091	-0.667	0.486	1.000
STRB-MmdW	-0.189	-0.765	0.388	0.988
PWD-BUSNA	1.087	0.387	1.787	< 0.001
STUF-BUSNA	0.945	0.245	1.645	< 0.001
STUS-BUSNA	0.159	-0.541	0.859	0.999
MmmW-BUSNA	0.656	0.079	1.232	0.013
SCHEST-BUSNA	-1.704	-2.404	-1.004	< 0.001
SIT-BUSNA	-0.126	-0.826	0.574	1.000
STRA-BUSNA	-0.589	-1.289	0.111	0.181
STRB-BUSNA	-0.687	-1.387	0.013	0.059
STUF-PWD	-0.142	-0.842	0.558	1.000
STUS-PWD	-0.928	-1.628	-0.228	0.002
MmmW-PWD	-0.431	-1.008	0.145	0.330
SCHEST-PWD	-2.791	-3.491	-2.091	< 0.001
SIT-PWD	-1.213	-1.913	-0.513	< 0.001
STRA-PWD	-1.676	-2.376	-0.976	< 0.001
STRB-PWD	-1.774	-2.474	-1.074	< 0.001
STUS-STUF	-0.786	-1.486	-0.086	0.015
MmmW-STUF	-0.289	-0.866	0.287	0.838
SCHEST-STUF	-2.649	-3.349	-1.949	< 0.001
SIT-STUF	-1.071	-1.771	-0.371	< 0.001
STRA-STUF	-1.534	-2.234	-0.834	< 0.001
STRB-STUF	-1.632	-2.332	-0.932	< 0.001
MmmW-STUS	0.497	-0.080	1.073	0.156
SCHEST-STUS	-1.863	-2.563	-1.163	< 0.001
SIT-STUS	-0.285	-0.985	0.415	0.949
STRA-STUS	-0.748	-1.448	-0.048	0.026
STRB-STUS	-0.846	-1.546	-0.146	0.006
SCHEST-MmmW	-2.360	-2.936	-1.783	< 0.001
SIT-MmmW	-0.782	-1.358	-0.205	< 0.001
STRA-MmmW	-1.245	-1.821	-0.668	< 0.001
STRB-MmmW	-1.343	-1.919	-0.766	< 0.001
SIT-SCHEST	1.578	0.878	2.278	< 0.001
STRA-SCHEST	1.115	0.415	1.815	< 0.001
STRB-SCHEST	1.017	0.317	1.717	< 0.001
STRA-SIT	-0.463	-1.163	0.237	0.510
STRB-SIT	-0.561	-1.261	0.139	0.237
STRB-STRA	-0.098	-0.798	0.602	1.000

I. Sperm tail length

Comparison	Difference in means	Lower Cl95%	Upper Cl95%	P_{adj}
BUSNA-MmdW	1.443	-0.888	3.774	0.605
PWD-MmdW	1.816	-0.515	4.147	0.274
STUF-MmdW	-0.789	-3.120	1.542	0.985
STUS-MmdW	2.008	-0.323	4.339	0.157
MmmW-MmdW	0.321	-1.370	2.012	1.000
SCHEST-MmdW	3.067	0.736	5.398	0.002
SIT-MmdW	3.473	1.142	5.804	< 0.001
STRA-MmdW	3.031	0.700	5.362	0.002
STRB-MmdW	1.898	-0.433	4.229	0.218
PWD-BUSNA	0.373	-2.457	3.203	1.000
STUF-BUSNA	-2.232	-5.062	0.598	0.258
STUS-BUSNA	0.565	-2.265	3.395	1.000
MmmW-BUSNA	-1.122	-3.453	1.209	0.869
SCHEST-BUSNA	1.624	-1.206	4.454	0.703
SIT-BUSNA	2.030	-0.800	4.860	0.390
STRA-BUSNA	1.588	-1.242	4.418	0.729
STRB-BUSNA	0.455	-2.375	3.285	1.000
STUF-PWD	-2.605	-5.435	0.225	0.099
STUS-PWD	0.192	-2.638	3.022	1.000
MmmW-PWD	-1.495	-3.826	0.836	0.555
SCHEST-PWD	1.251	-1.579	4.081	0.917
SIT-PWD	1.657	-1.173	4.487	0.678
STRA-PWD	1.215	-1.615	4.045	0.930
STRB-PWD	0.082	-2.748	2.912	1.000
STUS-STUF	2.797	-0.033	5.627	0.055
MmmW-STUF	1.110	-1.221	3.441	0.876
SCHEST-STUF	3.856	1.026	6.686	< 0.001
SIT-STUF	4.262	1.432	7.092	< 0.001
STRA-STUF	3.820	0.990	6.650	< 0.001
STRB-STUF	2.687	-0.143	5.517	0.078
MmmW-STUS	-1.687	-4.018	0.644	0.377
SCHEST-STUS	1.059	-1.771	3.889	0.970
SIT-STUS	1.465	-1.365	4.295	0.811
STRA-STUS	1.023	-1.807	3.853	0.976
STRB-STUS	-0.110	-2.940	2.720	1.000
SCHEST-MmmW	2.746	0.415	5.077	0.008
SIT-MmmW	3.152	0.821	5.483	< 0.001
STRA-MmmW	2.710	0.379	5.041	0.010
STRB-MmmW	1.577	-0.754	3.908	0.477
SIT-SCHEST	0.406	-2.424	3.236	1.000
STRA-SCHEST	-0.036	-2.866	2.794	1.000
STRB-SCHEST	-1.169	-3.999	1.661	0.945
STRA-SIT	-0.442	-3.272	2.388	1.000
STRB-SIT	-1.575	-4.405	1.255	0.738
STRB-STRA	-1.133	-3.963	1.697	0.954

J. Total sperm length

Comparison	Difference in means	Lower Cl95%	Upper Cl95%	P_{adj}
BUSNA-MmdW	2.771	0.252	5.290	0.019
PWD-MmdW	4.066	1.547	6.585	< 0.001
STUF-MmdW	0.628	-1.891	3.147	0.998
STUS-MmdW	2.928	0.409	5.447	0.010
MmmW-MmdW	1.958	0.130	3.785	0.025
SCHEST-MmdW	2.009	-0.510	4.528	0.244
SIT-MmdW	4.068	1.549	6.587	< 0.001
STRA-MmdW	3.289	0.770	5.808	0.002
STRB-MmdW	1.611	-0.908	4.130	0.559
PWD-BUSNA	1.295	-1.763	4.353	0.936
STUF-BUSNA	-2.143	-5.201	0.915	0.424
STUS-BUSNA	0.157	-2.901	3.215	1.000
MmmW-BUSNA	-0.813	-3.332	1.706	0.989
SCHEST-BUSNA	-0.762	-3.820	2.296	0.998
SIT-BUSNA	1.297	-1.761	4.355	0.935
STRA-BUSNA	0.518	-2.540	3.576	1.000
STRB-BUSNA	-1.160	-4.218	1.898	0.968
STUF-PWD	-3.438	-6.496	-0.380	0.015
STUS-PWD	-1.138	-4.196	1.920	0.971
MmmW-PWD	-2.108	-4.627	0.411	0.187
SCHEST-PWD	-2.057	-5.115	1.001	0.485
SIT-PWD	0.002	-3.056	3.060	1.000
STRA-PWD	-0.777	-3.835	2.281	0.998
STRB-PWD	-2.455	-5.513	0.603	0.235
STUS-STUF	2.300	-0.758	5.358	0.322
MmmW-STUF	1.330	-1.189	3.849	0.793
SCHEST-STUF	1.381	-1.677	4.439	0.907
SIT-STUF	3.440	0.382	6.498	0.015
STRA-STUF	2.661	-0.397	5.719	0.147
STRB-STUF	0.983	-2.075	4.041	0.989
MmmW-STUS	-0.970	-3.489	1.549	0.964
SCHEST-STUS	-0.919	-3.977	2.139	0.994
SIT-STUS	1.140	-1.918	4.198	0.971
STRA-STUS	0.361	-2.697	3.419	1.000
STRB-STUS	-1.317	-4.375	1.741	0.929
SCHEST-MmmW	0.051	-2.468	2.570	1.000
SIT-MmmW	2.110	-0.409	4.629	0.186
STRA-MmmW	1.331	-1.188	3.850	0.792
STRB-MmmW	-0.347	-2.866	2.172	1.000
SIT-SCHEST	2.059	-0.999	5.117	0.484
STRA-SCHEST	1.280	-1.778	4.338	0.940
STRB-SCHEST	-0.398	-3.456	2.660	1.000
STRA-SIT	-0.779	-3.837	2.279	0.998
STRB-SIT	-2.457	-5.515	0.601	0.234
STRB-STRA	-1.678	-4.736	1.380	0.754
